# Clonal Evolution of Acute Myeloid Leukemia with *CEBPA* Double Mutations after Long-Term Remission: Case Report and a Literature Review

**DOI:** 10.4274/tjh.galenos.2018.2018.0215

**Published:** 2019-05-03

**Authors:** Ying Li, Long Su

**Affiliations:** 1 Changchun Central Hospital, Clinic of Hematology, Changchun, China; 2 Jilin University the First Hospital, Clinic of Hematology, Changchun, China

**Keywords:** Acute myeloid leukemia, CEBPA mutations, Next-generation sequencing, Clonal evolution, Relapse

## To the Editor,

Mutations in the *CEBPA *gene occur in 7%-15% of all acute myeloid leukemia (AML) patients [1,2]. However, we found that the frequency of such mutation may be high in Chinese AML patients [3,4]. Although AML with *CEBPA* double mutations *(CEBPA*^dm^*)* indicates a favorable outcome, recent data show that more than 50% of patients finally relapsed when consolidated with chemotherapy alone [5]. Clonal evolution (CE) is an important factor for relapse [6]. However, studies discussing CE in AML patients with *CEBPA*^dm^ are limited [7,8]. Here, we report CE in two patients with *CEBPA*^dm^ determined by sensitive next-generation sequencing (NGS).

**Figure 1:**
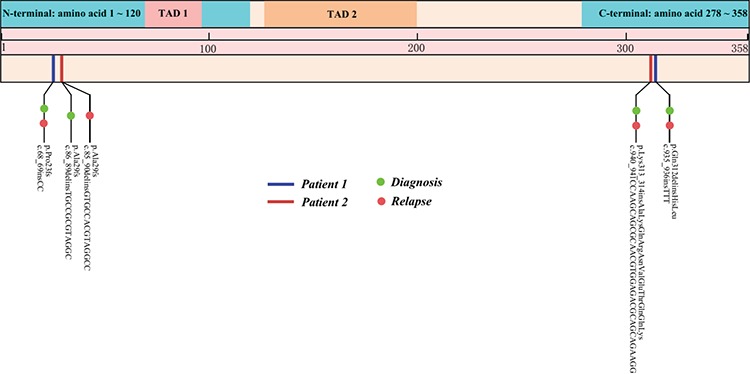
*CEBPA* gene mutations of these two patients at diagnosis and relapse.

Two female AML patients were diagnosed in our hospital in January 2012 and September 2013. Standard ‘3+7’ induction chemotherapy was administered. Both of them achieved CR after induction therapy. Patient 1 received consolidation therapy with one course of DA (daunorubicin + cytarabine), four courses of high-dose cytarabine (HD-Ara-C), and one course of DA. Patient 2 received consolidation therapy with three courses of HD-Ara-C and two courses of immunotherapy. After long-term remissions (63 and 40 months), they both relapsed. Cytogenetic and fusion gene analyses indicated no difference from diagnosis. NGS analysis indicated altered mutations sites of the *CEBPA* gene in Patient 2 (Figure 1). New co-occurring mutations emerged at relapse: *SETD2* mutation in Patient 1 and *WT1* mutation in Patient 2 (Table 1). After relapse, Patient 1 achieved CR with a DA regimen and Patient 2 refused treatment. 

The first report for CE in patients with *CEBPA*^dm^ included two patients [7]. In the first patient, the amino-terminal frame-shift mutation was duplicated and found on both alleles at relapse. In the second patient, the amino-terminal frame-shift mutation and a mutation in the fork region were found either alone or combined on the same allele, suggesting a subclone formation [7]. Another study reported CE in 22 patients; two of them lost mutations and none acquired new mutation at relapse [8]. Twenty patients harboring *CEBPA* mutations relapsed with identical mutation patterns; three of them had a second relapse that also exhibited the same patterns as their initial diagnosis and first relapse [8]. Two patients had concomitant *FLT3*-ITD mutations at diagnosis and one was lost at relapse. Two patients acquired *FLT3*-TKD mutations at relapse. N-RAS mutations were detected in three patients at diagnosis and two of them retained the identical mutation at relapse [8]. In this case report, we found mutation site alteration in the *CEBPA* gene and two newly emerged co-occurring mutations.

CE of patients with *CEBPA*^dm^ can be summarized as follows: 1) allele alteration of *CEBPA* gene: acquire or lose mutation site in allele; 2) mutation site alteration in *CEBPA* gene: acquire or lose mutation site in *CEBPA* gene other than allele; 3) co-occurring mutation alteration: acquire or lose co-occurring mutation. One issue that needs to be resolved is the relationship between time and CE after CR. In this case report, these two patients relapsed after long-term remissions, and new co-occurring mutations emerged in both of them. Hence, whether late relapse is associated with new co-occurring mutations is unknown.

## Figures and Tables

**Table 1:**
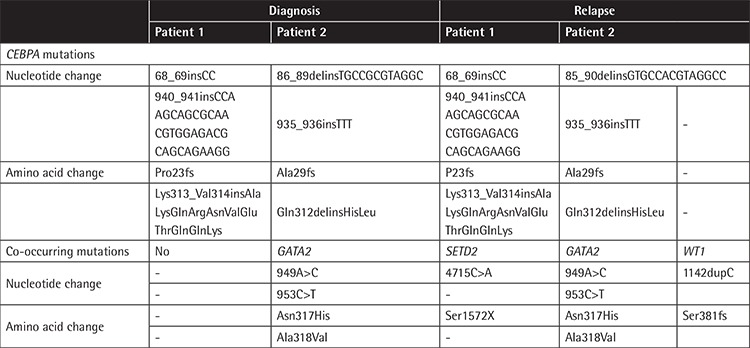
*CEBPA* and co-occurring mutations at diagnosis and relapse.
